# MDM2 inhibitors in cancer immunotherapy: Current status and perspective

**DOI:** 10.1016/j.gendis.2024.101279

**Published:** 2024-03-28

**Authors:** Qinru Zeng, Shaocheng Zeng, Xiaofeng Dai, Yun Ding, Chunye Huang, Ruiwen Ruan, Jianping Xiong, Xiaomei Tang, Jun Deng

**Affiliations:** a Department of Oncology, The First Affiliated Hospital, Jiangxi Medical College, Nanchang University, Nanchang, Jiangxi 330006, China; b Jiangxi Key Laboratory for Individual Cancer Therapy, Nanchang, Jiangxi 330006, China; c Department of Oncology, Jiangxi Chest Hospital, Nanchang, Jiangxi 330006, China; d Postdoctoral Innovation Practice Base, The First Affiliated Hospital of Nanchang University, Nanchang, Jiangxi 330006, China

**Keywords:** Immune hyperprogression, Immune microenvironment, Immunotherapy, MDM2, MDM2 inhibitors, p53

## Abstract

Murine double minute 2 (MDM2) plays an essential role in the cell cycle, apoptosis, DNA repair, and oncogene activation through p53-dependent and p53-independent signaling pathways. Several preclinical studies have shown that MDM2 is involved in tumor immune evasion. Therefore, MDM2-based regulation of tumor cell-intrinsic immunoregulation and the immune microenvironment has attracted increasing research attention. In recent years, immune checkpoint inhibitors targeting PD-1/PD-L1 have been widely used in the clinic. However, the effectiveness of a single agent is only approximately 20%–40%, which may be related to primary and secondary drug resistance caused by the dysregulation of oncoproteins. Here, we reviewed the role of MDM2 in regulating the immune microenvironment, tumor immune evasion, and hyperprogression during immunotherapy. In addition, we summarized preclinical and clinical findings on the use of MDM2 inhibitors in combination with immunotherapy in tumors with MDM2 overexpression or amplification. The results reveal that the inhibition of MDM2 could be a promising strategy for enhancing immunotherapy.

## Introduction

**Figure 1:**
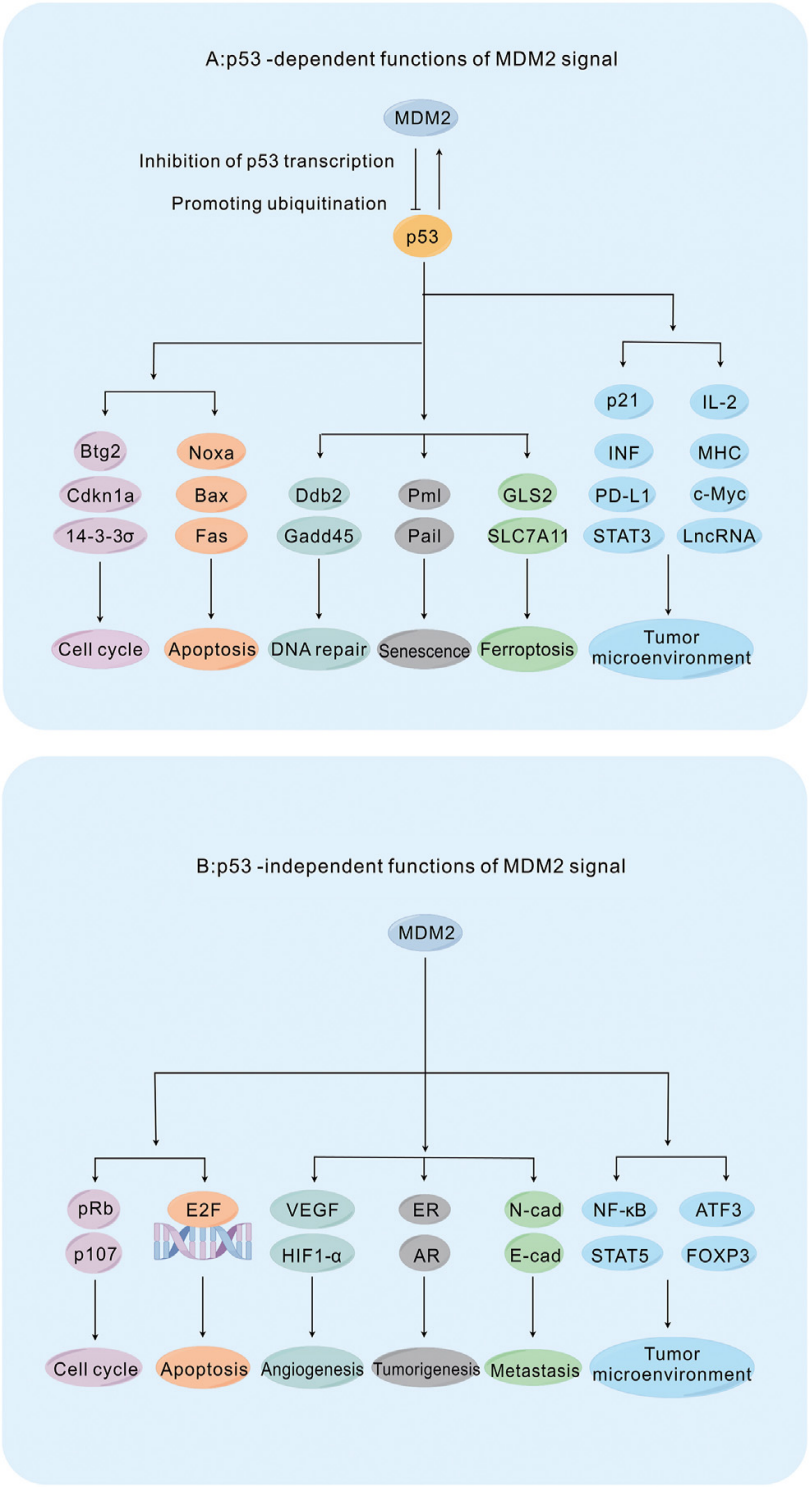
MDM2 signaling pathway. **(A)** MDM2 proteins degrade and inactivate p53 through ubiquitination and inhibition of p53 transcription, thereby regulating various physiological and pathological processes such as cell cycle, apoptosis, DNA damage repair, senescence, iron death, and TME. **(B)** MDM2 protein regulates various physiological and pathological processes with p53-independent MDM2 pathway signaling pathways such as cell cycle, apoptosis, angiogenesis, tumorigenesis, metastasis, and TME. MDM2, murine double minute 2; TME, tumor microenvironment; Cdkn1a, cyclin-dependent kinase inhibitor 1A; Btg2, B cell translocation gene 2, anti-proliferative; Bax, BCL-2-associated X protein; Noxa, superoxide-generating NADPH oxidase; Fas, a classic apoptosis modulator; Gadd45a, growth arrest and DNA damage-inducible 45a; Ddb2, damage-specific DNA binding protein 2; Pml, promyelocytic leukemia; Pai1, plasminogen activator inhibitor; GLS2, glutaminase 2; SLC7A11, solute carrier family 7 member 11; STAT3, signal transducer and activator of transcription 5; lncRNA, long non-coding RNA; p107, Rb family members; E2F, elongation factor 2; VEGF, vascular epithelial growth factor; HIF1-α, hypoxia-inducible factor-alpha; E-cad, E-cadherin; N-cad, N-cadherin; ER, estrogen receptor; AR, androgen receptor; ATF3, activating transcription factor 3.

Murine double minute 2 (MDM2) was first discovered in 1987 in the 3T3-DM cell line. MDM2 overexpression in NIH3T3 and Rat2 cells was shown to lead to the development of cancer,^1^ and MDM2 was subsequently shown to be a major negative regulator of p53.^2^ The MDM2 protein contains four functional regions, from the N-terminus to the C-terminus: the p53-binding region, the acidic region, the zinc-finger region, and the RING domain.^2^ The RING and zinc-finger regions are key to the E3 ligase activity of MDM2.^3^ MDM2 mainly functions via p53-dependent pathways. The level and activity of wild-type p53 (wtp53) increases in response to various cellular stresses, such as DNA damage, hypoxia, and oncogenic activation. Consequently, wtp53 acts as a transcription factor and tumor suppressor to repair damaged cells, prevent cellular carcinogenesis, and eradicate malignant cells.^4^ Activation of wtp53 induces the expression of MDM2,^5^^,^^6^ whereas MDM2 inhibits p53 by blocking its transcriptional activity or promoting its ubiquitination and degradation.^3^^,^^7^ Functional inactivation of p53 occurs when MDM2 is overexpressed or amplified, as revealed in a study showing that p53 is inactivated in 29 of 33 MDM2-amplified tumors.^8^^,^^9^ MDM2 indirectly regulates Cdkn1a, Fas, Noxa, Gadd45a, and other key molecules by inactivating p53, thereby regulating the cell cycle, apoptosis, and DNA repair (Fig. 1A).^10^ In addition, MDM2 can directly regulate molecules such as pRB, p107, E2F, and HIF1-α/VEGFA in a p53-independent pathway, thus regulating the cell cycle, apoptosis, and angiogenesis (Fig. 1B). Tumor growth, metastasis, and immune escape increase when MDM2 is overexpressed or amplified. Moreover, MDM2-induced immune escape of tumor cells is associated with the overexpression of PD-1/PD-L1, low availability of neoantigens, and down-regulation of major histocompatibility complex (MHC-I and MHC-II) (Fig. 1A).^2^^,^^11^, ^12^, ^13^, ^14^, ^15^

MDM2 is involved in the regulation of the tumor microenvironment (TME) in p53-dependent and p53-independent manners. MDM2-mediated inhibition of p53 not only increases T-cell proliferation and activation through interferon (IFN) and interleukin (IL)-2/T-cell receptor signaling but also inhibits Treg T-cell differentiation and promotes macrophage M1 polarization through the regulation of FOXP3, c-Myc, and c-Maf (Fig. 1A). MDM2 also regulates STAT5 and NF-κB to increase T-cell activity and proliferation in a p53-independent manner (Fig. 1B). However, in other studies, MDM2 has been found to promote immune evasion, which is possibly due to the tumor heterogeneity triggered by differences in individuals, cancer types, epigenetic inheritance, signaling pathways, and other factors. Despite the promising anti-tumor effects of MDM2 inhibitors in preclinical studies, their efficacy in clinical studies is unsatisfactory, and adverse effects limit their further application in the clinic. Therefore, further studies are required to improve the efficacy and safety of MDM2 inhibitor therapy. Clinical translational studies on MDM2 inhibitors in combination with immunotherapy offer hope. Combining these two approaches enhances tumor resistance to therapy, improves the anti-tumor efficacy of drugs, and does not appear to exacerbate the adverse reactions associated with MDM2 inhibitors. Therefore, this review discusses the progress of MDM2 inhibitors and their combination for immunotherapy.

In recent years, the development of immune checkpoint inhibitors (ICIs), such as anti-cytotoxic T lymphocyte-associated protein 4 and anti-PD-1/PD-L1, has led to significant breakthroughs in anti-tumor therapy.^16^ However, the response rates of ICIs in controlling tumors remain low. For instance, in patients with melanoma, the efficacy of ipilimumab (anti-cytotoxic T lymphocyte-associated protein 4) is only approximately 21%.^17^ Similarly, in many solid tumors, such as urothelial carcinoma, non-small cell lung cancer, and Merkel cell carcinoma, the effectiveness of PD-1/PD-L1 inhibitors ranges from 20% to 40%. Moreover, ICI therapy failure often corresponds to non-response (primary resistance) and tumor progression or recurrence (secondary resistance).^16^ The primary reasons for resistance include low mutation burden, neoantigen loss, chromatin remodeling, dysfunctional or exhausted T cells, gene amplification, and multiple immune regulatory signaling pathway dysregulation.^18^ MDM2 overexpression or amplification leads to the inactivation of p53, resistance to ICIs, and hyperprogression during immunotherapy. Therefore, understanding the role of MDM2 in regulating the TME and immune response may help improve the efficacy of immunotherapy.

## MDM2 regulates the immune microenvironment in p53-dependent and p53-independent manners

Previous studies have suggested that tumors function in isolation. However, recent studies have indicated that the TME plays a crucial role in tumor treatment resistance and immune evasion. The TME mainly consists of various immune cells, stromal cells, extracellular matrix, secreted factors, and vascular systems,^19^ and it exhibits tumor suppression or support depending on the percentage of immunosuppressive and immunosupportive components, which correlates with organ and tumor characteristics, tumor stage, and patient-specific factors.^20^ However, in most advanced solid tumors, the TME is composed mainly of immunosuppressive components. Loss and functional inactivation of p53 drive the transition of the TME toward a pro-tumorigenic inflammatory state, and the reactivation of p53 can reverse immune suppression.^21^ MDM2 is involved in the regulation of the TME mainly through the negative regulation of p53, although several studies have shown that it is involved through p53-independent signaling pathways.

### CD4^+^/CD8^+^ T cells

CD8^+^ T cells activate cytotoxic T lymphocytes (CTLs) after binding to MHC-I on the membranes of antigen-presenting cells and tumor cells through T-cell receptors. Subsequently, CTLs exert cytotoxic effects by releasing granzyme B and perforin or secreting cytokines (*e.g.*, IFN) and FASL-FAS, ultimately resulting in the death of tumor cells.^22^, ^23^, ^24^ CD4^+^ cells can differentiate into diverse T helper (Th) cell subsets, including Th1, Th2, and Th17 cells.^25^ Th1 cells are critical for assisting CTL activation,^26^ which can also directly eliminate cancer cells through the secretion of IFN-γ and tumor necrosis factor-alpha (TNF-α).^20^ Notably, Th2 cells promote tumor progression.^27^ When T cells are dysfunctional or depleted, tumors undergo immune escape.^22^ Notably, MDM2 is involved in regulating CD8^+^ and CD4^+^ T cells.

**Figure 2:**
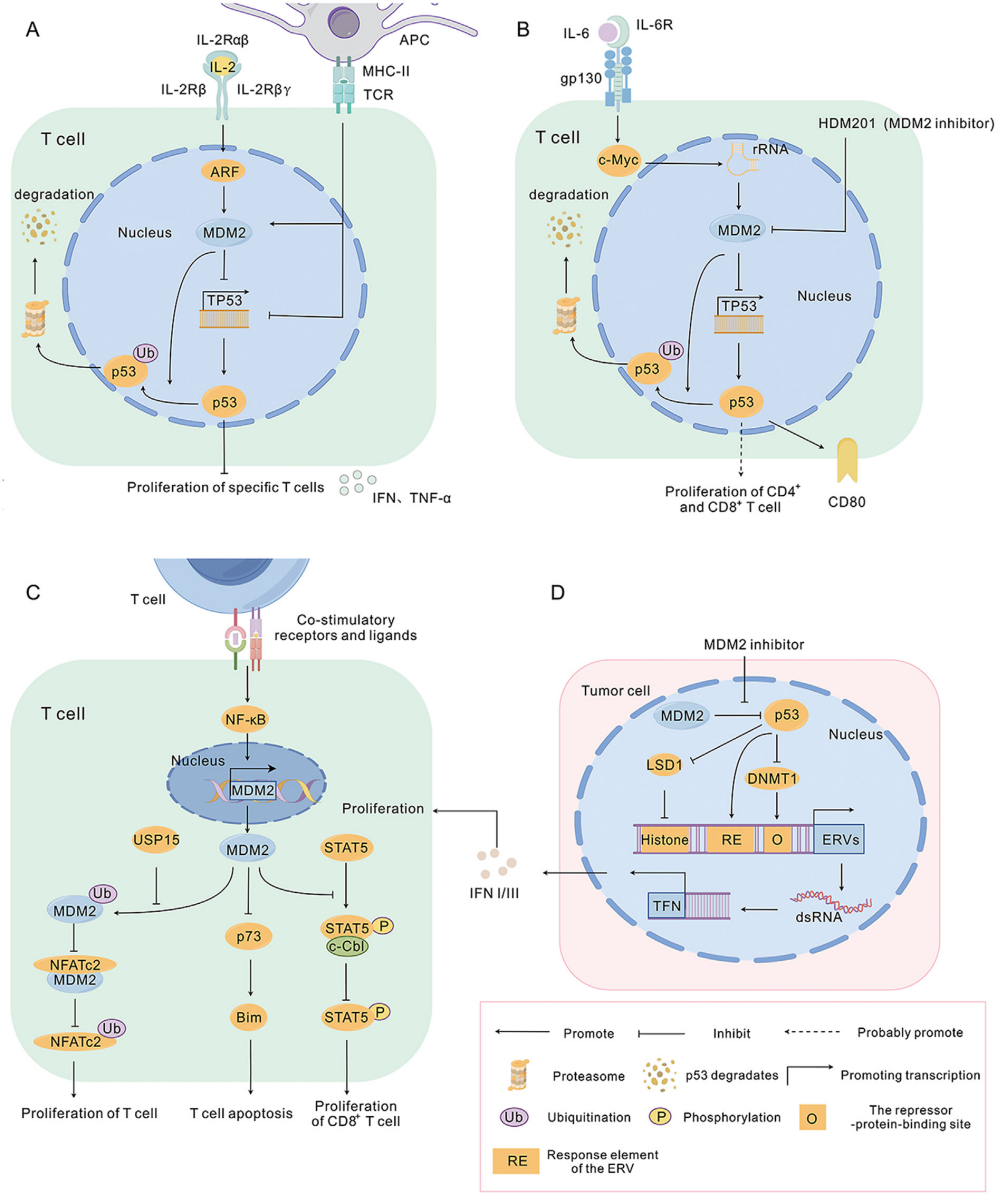
MDM2 in T cells. **(A)** IL-2 inhibits specific T cell proliferation by reducing ubiquitination of p53 through ARF inhibition of MDM2. When T-cell receptors are stimulated, they down-regulate p53 levels and promote the function of IL-2 on specific T cell proliferation. Activated T cells secrete IFN and TNF-α. **(B)** MDM2 promotes the formation of ribonucleoprotein-MDM2 that degraded p53 through IL-6-mediated ribosomal RNA transcription, which may lead to the inhibition of T cell proliferation. The MDM2 inhibitor HDM201 up-regulates p53 expression and activity, resulting in increased expression of the T-cell co-stimulatory factor CD80. **(C)** USP15 assists MDM2 ubiquitination to degrade NFATc2, which in turn inhibits T cell proliferation and activation. NF-κB promotes the transcription of MDM2 from the P1 promoter when T cells are co-stimulated, and up-regulated MDM2 inhibits Bim by suppressing the transcription of p73, which reduces T cell apoptosis. MDM2 aids STAT5 phosphorylation by inhibiting STAT5 degradation by c-Cbl, thereby promoting T cell survival. **(D)** MDM2 inhibitor-activated p53 binds to the promoter of ERV and inhibits the deterrents DNMT1 and LSD1, which promotes an increase in ERV transcription and induces interferon (INF I/III) production. MDM2, murine double minute 2; IFN, interferon; TNF-α, tumor necrosis factor-alpha; NFATc2, nuclear factor of activated T-cells, cytoplasmic 2; USP15, deubiquitinase 15; Bim, apoptotic protein; NF-kB, nuclear factor-κB; STAT5, signal transducer and activator of transcription 5; c-Cbl, an E3 ligase; ATF3, activating transcription factor 3; ERV, endogenous retroviruses; DNMT1, DNA methyltransferase 1; LSD1, histone demethylase 1.

MDM2 has been suggested to exert an inhibitory effect on T cells via a p53-dependent pathway. A diminished capacity of T cells to effectively eliminate tumor cells was observed in ovarian cancer cell lines with high expression of MDM2.^26^ Tumors treated with the MDM2 inhibitor AMG-232 showed enhanced anti-tumor effects mediated by T cells, concomitant with reduced IL-6 secretion.^28^ IL-6 impacts the expression of cytotoxic CD4^+^ T cells and the activation of CTLs by inhibiting IL-12 and IFN-γ.^29^ Moreover, IL-6 augments ribosomal RNA transcription by up-regulating c-Myc expression, which increases the degradation of p53 in the ribonucleoprotein MDM2 (Fig. 2B).^30^

We speculate that IL-6 may mediate immunosuppressive effects through the p53-dependent MDM2 pathway. Activated p53, facilitated by MDM2 inhibitors, binds to the promoters of endogenous retroviruses, leading to the inhibition of the epigenetic repressors DNA methyltransferase 1 and histone demethylase 1. This culminates in increased transcription of endogenous retroviruses and the formation of double-stranded RNA, subsequently triggering the production of IFN. IFN is one of the regulators of the T-cell antitumor response (Fig. 2D),^31^ which can further recruit T cells and ultimately inhibit the immune escape of tumor cells.^32^ In a wtp53 colon cancer model, the MDM2 inhibitor HDM201 up-regulated the expression and activity of p53. p53 induces the expression of the T-cell co-stimulatory factor CD80, thereby increasing the proportion of CD8^+^ T-cell subpopulations and the CD8^+^ T/Treg cell ratio. However, when p53 is knocked out, the expression of CD80 in the tumor is blocked and the anti-tumor effect of HDM201 is weakened (Fig. 2B).^33^ The MDM2 inhibitor ALRN-6924 also enhances the IFN signaling pathway by activating p53 and promoting cytotoxic CD8^+^ T-cell infiltration in melanoma patients.^32^ In wtp53 tumors, the MDM2 inhibitor APG-115 increases CD4^+^ T cell activity and CD4^+^ and CD8^+^ T-cell infiltration through the activation of p53, whereas APG-115, in combination with an anti-PD-1 inhibitor, synergistically promotes significant infiltration of toxic CD8^+^ T cells in both cellular and mouse models.^34^

Moreover, several studies have shown that MDM2 regulates T cells through p53-independent MDM2 signaling pathways. USP15 regulates the stability and activity of MDM2 in T cells and tumor cells. In melanoma and colorectal cell lines, deletion of USP15 induces MDM2 auto-ubiquitination and degradation and USP15 negatively regulates the T-cell transcription factor NFATc2 by decreasing MDM2 auto-ubiquitination and degradation, thereby inhibiting T-cell proliferation and activation (Fig. 2C).^35^

However, MDM2 inhibits T cell apoptosis and promotes T cell proliferation and activation. In activated T cells, binding of the activated transcription factor NF-κB to the P1 promoter of MDM2 increases MDM2 transcription, and then up-regulated MDM2 impedes apoptosis mediated by the apoptotic protein Bim by inhibiting the transcription of p73. In addition, in p53-deficient T cells, the MDM2 inhibitor chalcone leads to apoptosis of activated T cells (Fig. 2C).^36^

When T-cell receptors are unstimulated, IL-2 inhibits CD4^+^ T-cell proliferation by inducing p53 expression in CD4^+^ T cells, which may be associated with increased IL-2-mediated expression of the tumor suppressor ARF. ARF inhibits MDM2 ubiquitination of p53 by interacting with the zinc finger region and RING structural domain of MDM2.^37^ Elevated p53 levels inhibit T-cell proliferation, which may prevent the proliferation of nonspecific T cells.^38^ When T-cell receptors are stimulated, MDM2 expression is increased. Consequently, the level of p53 decreases in CD4^+^ T cells, thereby promoting IL-2-mediated proliferation of antigen-specific CD4^+^ T cells (Fig. 2A).^31^ In contrast, the MDM2 inhibitor Nutlin 3a inhibits antigen-specific CD4^+^ T-cell proliferation.

A subsequent study presented new insights into the role of MDM2 in CD8^+^ T-cell activation and proliferation. The authors observed that upon binding of MDM2 to STAT5 in CD8^+^ T cells, STAT5 was protected from degradation by c-Cbl, resulting in enhanced stability of STAT5, which led to the proliferation and activation of CD8^+^ T cells (Fig. 2B). This process was independent of the status of p53, which was validated by *in vivo* and *in vitro* experiments. Thus, the authors proposed that MDM2 is a key component of CD8^+^ T-cell survival and activation.^39^ Interestingly, the authors suggested that APG-115 could act as a sensitizer of MDM2; however, this hypothesis still needs to be confirmed.

In summary, T-cell regulation by MDM2 or MDM2 inhibitors is complex and bidirectional. First, this seemingly conflicting result may be related to two different MDM2 inhibitors that inhibit the E3 ubiquitin ligase activity of MDM2 and impede the mutual binding of MDM2 and p53.^35^ Different MDM2 inhibitors have different effects on T cell activation. Second, this result may be related to different regulatory pathways. For example, MDM2 has been shown to inhibit T-cell proliferation and activation by negatively regulating NFATc2, whereas another report showed that MDM2 promotes CD8^+^ T cell proliferation and activation by stabilizing STAT5.^35^^,^^39^ Meanwhile, MDM2-overexpressing tumors are mainly “cold tumors”. Data from existing preclinical and clinical studies revealed that MDM2 inhibitors have not been shown to impair the efficacy of immunotherapy. In contrast, MDM2 inhibitors exhibit synergistic effects when used in combination with immunotherapy. Therefore, we believe that MDM2 exerts a predominantly immunosuppressive effect.

### Natural killer cells

Natural killer (NK) cells kill tumor cells through death receptor interactions, perforin/enzyme-mediated cytotoxicity, and the secretion of cytokines, such as IFN-γ.^40^^,^^41^ NK cells differ from CD8^+^ T cells in that NK cell function is influenced by activating receptors (*e.g.*, NKG2D and DNAx (*e.g.*, DNAM-1 receptor)) and inhibitory receptors.^42^, ^43^, ^44^

In some tumors, NK and memory NK cells become dysfunctional, a process associated with the MDM2-p53 pathway. In a mouse model of hepatocellular carcinoma, activated p53 was found to increase NK cell recognition and infiltration through a decay-inducing program, which was not dependent on NKG2D but rather correlated with cytokines (IL-12, IL-15, IL-18, CCL2, and CCL3) produced by activated p53. Subsequent studies have shown that p53 transcriptionally activates CCL2 to promote the mass recruitment of NK cells to senescent tumors, leading to the elimination of tumor cells that express NKG2D ligands on the surface.^45^^,^^46^ In neuroblastoma, ligands for the NK cell receptors NKGD2 and DNAM-1 NK-AR are inhibited and NK cell-mediated innate immunity is suppressed,^44^ which has been reported to be associated with the inactivation of p53. MYCN amplification, a poor prognostic indicator of neuroblastoma, inhibits p53 function by inducing MDM2.^47^ Therefore, the MDM2 inhibitor Nutlin-3a can be used to activate p53, which subsequently induces the expression of PVR, a ligand of the NK cell receptor DNAM-1, resulting in enhanced NK cell killing by neuroblastoma.^48^

IL-15 is essential for the activation, proliferation, and cytotoxic effects of NK cells. Subcutaneous injection or intravenous administration of IL-15 in metastatic malignant tumors promotes NK cell activation.^49^ Decreased p53 activation and degradation by MDM2 inhibition in mouse melanoma promotes increased IL-15 transcription.^50^ Therefore, we suggest that IL-15-induced activation of NK cells is mediated by the MDM2–p53 pathway. However, continued stimulation of NK-cell activation by IL-15 leads to severe large granular lymphocytic leukemia.^51^ The underlying mechanism may be that IL-15 causes large granular lymphocytic leukemia by inducing Akt to mediate MDM2 expression, thereby inhibiting the action of the proapoptotic molecule Bid (a member of the Bcl-2 family).^52^^,^^53^

### Dendritic cells

Dendritic cells (DCs) are functionally specialized antigen-presenting cells that play an indispensable role in suppressing tumor immune escape.^54^^,^^55^ DCs activate CD8^+^ T cells by recognizing and presenting antigens specific to tumor cells, promoting the differentiation of CD4^+^ T cells into a variety of Th cells, and secreting cytokines such as IL-12.^56^, ^57^, ^58^ Moreover, DCs can mediate the generation of Tregs.^59^^,^^60^ Tumors can transform DCs into FOXP3^+^ Tregs, and DCs can also secrete IL-10 to induce the transformation of T cells into Tregs,^61^ thus revealing that DCs can promote tumor development. DCs often exhibit functional impairment and tolerance in the TME, thus exhibiting plasticity.^62^

The activation of p53 promotes DC typing and anti-tumor effects.^33^^,^^63^^,^^64^ Bone marrow-derived angiogenic progenitor cells are a type of DC produced by the bone marrow of mice cultured with granulocyte-macrophage colony-stimulating factor. The presence of wtp53 is critical for bone marrow-derived angiogenic progenitor cells to induce toxicity in tumors and exert immunopreventive effects. When wtp53 is absent in bone marrow-derived angiogenic progenitor cells, the signaling pathway by which bone marrow-derived angiogenic progenitor cells induce CD8^+^ T cell activation in CTLs is impaired. The main mechanism is that the deletion of wtp53 prevents it from functioning as a nuclear transcription factor to bind to the IL-12 p40 promoter, leading to a decrease in the production of IL-12. Therefore, the activation of CTLs and NK cells and the production of cytokines, such as IFN-γ, are suppressed.^55^^,^^65^

MDM2 is frequently amplified or overexpressed to inactivate p53 in some tumor cells, leading to the impairment of the anti-tumor effect of DCs. Thus, MDM2 inhibitors may boost the tumor immune response by activating DCs. Studies have confirmed that the MDM2 inhibitor Nutlin-3 increases DC proliferation and maturation by stabilizing and activating p53, and the mechanism may be related to the involvement of p53 in pathways such as NF-κB, Bcl-2, FOXO, and Notch-1.^64^^,^^66^ In colorectal cancer, the MDM2 inhibitor HDM201 increases the number of CD103^+^ DCs through the activation of p53, resulting in enhanced cytotoxicity of CD8^+^ T cells.^33^ Therefore, the intrinsic mechanism of DC dysfunction and tolerance to progenitors is related to the MDM2-p53 pathway.

### Regulatory T cells and tumor-associated macrophages

Regulatory T cells (Tregs) and tumor-associated macrophages (TAMs) are the predominant cells that promote immune escape from tumors. Tregs characterized by FOXP3^+^ CD25^+^ CD4^+^ expression promote tumor immune evasion by suppressing the immune responses of CD8^+^ and CD4^+^ T cells.^67^^,^^68^ FOXP3 is a key transcription factor in regulating Tregs.^69^^,^^70^ In the TME, Tregs assist tumor cells in immune escape through the suppression of innate and adaptive immunity, which appears to be associated with the p53-dependent MDM2 signaling pathway. p53 stimulates Treg differentiation through direct interaction with STAT-5, and MDM2 can modulate this process.^71^ In a p53-deficient KRAS-induced pancreatic cancer model, p53-deficient tumors promoted immune tolerance by recruiting Treg cells.^67^ In addition. activation of T-cell receptor/CD28 signaling in Tregs mediates MDM2 up-regulation, and FOXP3 can be stabilized at post-translational levels by MDM2 non-degradative ubiquitylation modification; thus, MDM2 up-regulation promotes the proliferation and activity of Tregs.^72^ Subsequently, in esophageal cancer cells, researchers observed that long non-coding RNA MEG3 promotes ubiquitination degradation of p53 by regulating MDM2 expression, which leads to down-regulation of miR-149-3p, and in turn, promotes FOXP3^+^ expression. As a result, the differentiation of Tregs was increased.^73^

Monocytes from the peripheral blood recruited to the TME are rapidly transformed into TAMs. TAMs account for 50% of the tumor parenchyma and can be polarized toward both anti-inflammatory M1 and immunosuppressive M2 subgroups.^62^ M1 macrophages promote the secretion of anti-inflammatory and immunostimulatory factors, such as IL-12, and assist in the Th1 immune response, whereas M2 macrophages promote epithelial–mesenchymal transition, angiogenesis, and immune escape of tumor cells. Under the stimulation of tumor progression, chemokines, and cytokines, such as IL-4 and IL-10, TAMs in the TME are mainly polarized toward M2 macrophages.^74^

**Figure 3:**
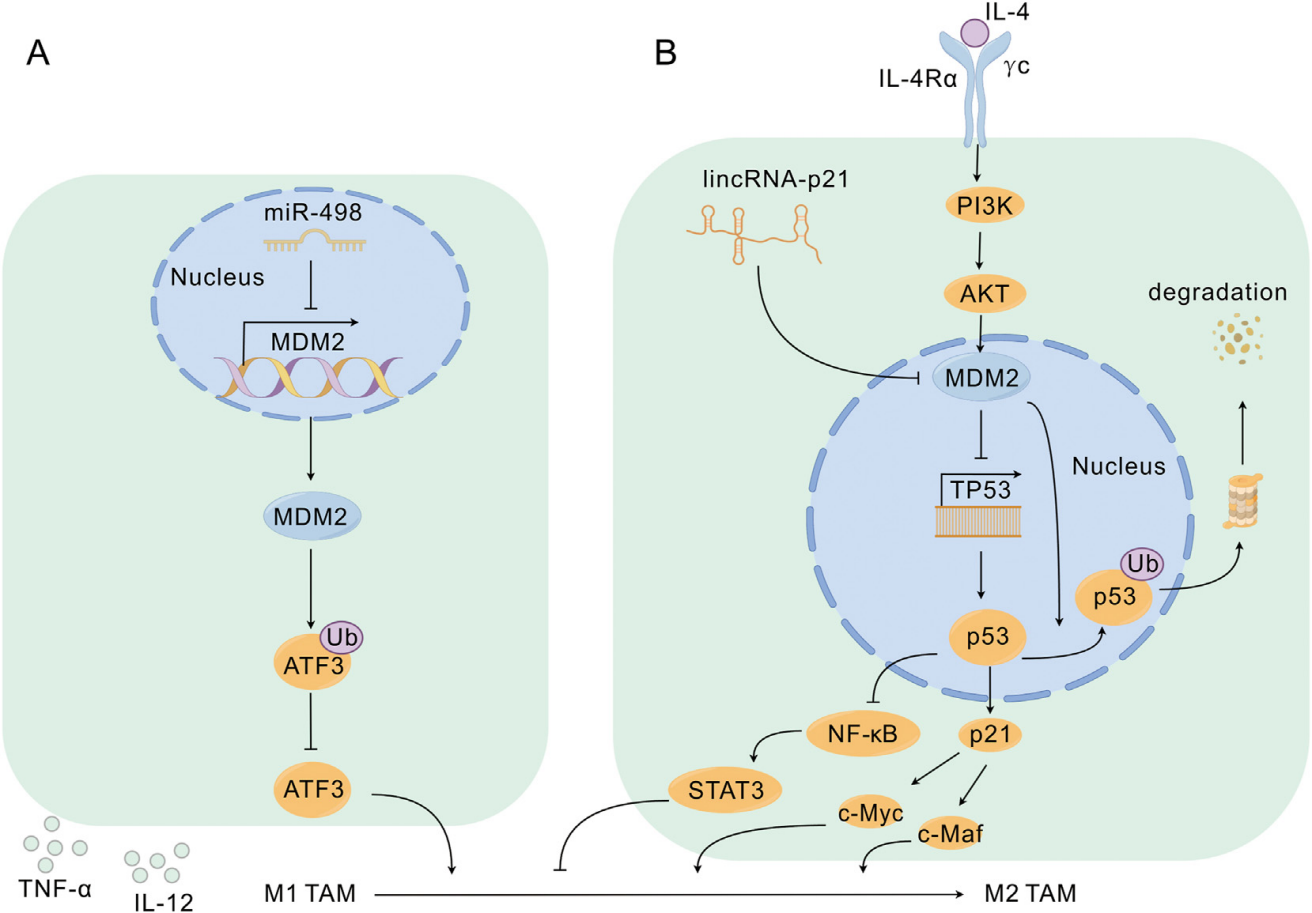
MDM2 in TAM. **(A)** MDM2 inhibits MDM2-mediated ubiquitination of ATF3 through miR-498, thereby inhibiting M1 to M2 polarization. M1 secretes immunostimulatory factors IL-12, TNF-α, *etc*. **(B)** MDM2 promotes p53 degradation through IL-4-dependent activation of the PI3K/Akt pathway, resulting in the down-regulation of p21, which leads to the up-regulation of the key M2 factors c-Myc and c-Maf. Inhibition of MDM2 by the up-regulated lincRNA-p21 causes the up-regulation of p53, leading to the inhibition of the NF-κB-STAT3 loop, which facilitates the polarization of M1 to M2. MDM2, murine double minute 2; lincRNA-p21, long intergenic non-coding RNA p21; miR-498, microRNA-498; PI3K, phosphatidylinositol 3-kinase; Akt, protein kinase B; TNF-α, tumor necrosis factor-alpha; NF-kB, nuclear factor-κB.

In breast cancer with high CD44 expression, TAMs release CCL8 to activate MDM2-p53 signaling and promote CD44 expression, thereby inducing tumor cell detachment, which facilitates breast cancer invasion and metastasis. In addition, the region downstream of MAPK/p38 is involved in CD44-dependent adhesive detachment.^75^ Therefore, we hypothesized that TAMs regulate this process through the MDM2-p53-MAPK-p38 axis, which warrants further exploration. Overexpression of MDM2 in gastric, colon, and breast cancers has been observed to regulate TAM polarization toward M2 to participate in tumor immune escape,^76^^,^^77^ and p53 serves as a transcriptional repressor of M2 polarization.^78^ IL-4 stabilizes MDM2 in macrophages by promoting MDM2 phosphorylation through the activation of PI3K/Akt, which increases the ubiquitination of p53 (Fig. 3B). This leads to the increased ubiquitination of p53. Consequently, in M2 macrophages, p53 levels remain low, thereby promoting M2 polarization. Further studies have shown that M2 polarization is inhibited by MDM2 inhibitors. APG-115 up-regulated the expression of wtp53 and p21 by affecting the p53-dependent MDM2 interaction, which led to the down-regulation of the key M2 factors c-Myc and c-Maf. Consequently, M2 polarizes toward M1^29^.

In addition, in mouse hepatic stellate cells, p53-expressing hepatic stellate cells were shown to induce senescence and a senescence-associated secretory phenotype, promote M1 polarization, and inhibit p53-expressing hepatic stellate cell-mediated macrophage polarization toward M2.^79^ In mouse models of myeloid lineage-specific p53 activation, p53 activation inhibits M2 macrophage polarization and c-Myc down-regulation.^80^ In lung and pancreatic adenocarcinomas, p53 deletion enhances the secretion of chemokines such as CCL11, CXCL1, CXCL5, CCL3, and M−CS from tumor cells, which increases macrophage infiltration.^67^

In esophageal cancer, miR-498 reduces MDM2 expression by binding to the MDM gene, MDM2 mediates attenuated ubiquitination of ATF3, and up-regulated ATF3 reduces macrophage autophagy and inhibits macrophage M2 polarization (Fig. 3A).^81^ In contrast, in breast cancer, up-regulated long-stranded intergenic non-coding RNA-p21 protects p53 from degradation by suppressing MDM2 expression, leading to inhibition of the NF-κB-STAT3 loop and promotion of M1 to M2 polarization (Fig. 3B).^82^ This is mainly due to the different roles of miR-498 and long-stranded intergenic non-coding RNA-p21 in tumors, with the former serving as a tumor suppressor and the latter acting as a tumor promoter.

## MDM2 and tumor immune evasion

**Figure 4:**
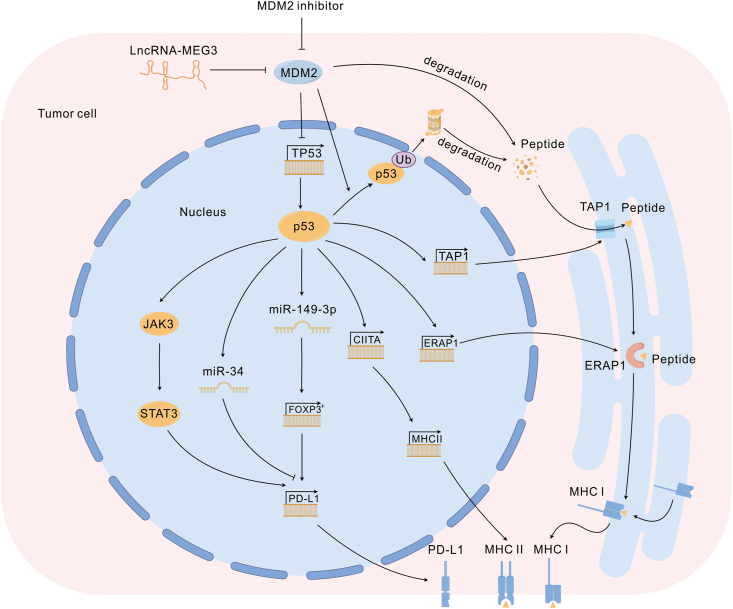
MDM2 in tumor cells. Antigenic peptides produced by the degradation of MDM2 with p53 in tumor cells can bind to MHC and activate T cells. MDM2 inhibitor up-regulates p53 levels and further promotes MHC expression on the tumor cell surface by activating TAP1, ERAP1, and CIITA. p53 up-regulated by MDM 2 inhibitor can activate JAK3/STAT3 signaling and miR-34 levels, and JAK3/STAT3 signaling can activate PD-L1 expression, but opposite miR-34. In addition, lncRNA MEG3 up-regulated miR-149-3p expression through the MDM2-p53 axis and further inhibited FOXP3 expression, activating PD-L1 transcription and directly inhibiting CD8^+^ T cell activity in the TME. MDM2, murine double minute 2; TAP1, transporter associated with antigen processing 1; ERAP1, endoplasmic reticulum aminopeptidase 1; CIITA, class II transactivator; JAK3, Janus kinase 3; STAT3, signal transducer and activator of transcription 3; miR, microRNA; FOXP3, forkhead box P3; lncRNA, long non-coding RNA. The schematic figure was drawn by Figdraw (www.figdraw.com).

Tumor immune escape refers to the phenomenon of tumor cells evading the recognition and attack of the immune system through various mechanisms to grow and metastasize, and it represents an essential strategy for tumor survival and development.^83^ Tumor immune escape is induced by various factors, including the low immunogenicity of tumor cells, recognition of tumor-specific antibodies as self-antigens, modulation of tumor surface antigens, tumor-induced immunity zones, and tumor-induced immunosuppression, which are the most widely investigated mechanisms. Tumor-induced immunosuppression occurs via two main pathways. The first is to reduce the immune tolerance of tumor cells by inducing the accumulation of immunosuppressive cells around the tumor and secreting immunosuppressive factors that inactivate CTLs and NK cells,^84^^,^^85^ while the second involves the induction of the expression of immunosuppressive molecules or their receptors, including immune checkpoints such as PD-L1/PD-1, CTLA4, and MHC, which can inhibit the activation of effector T lymphocytes, ultimately leading to tumor immune escape. Tumor cells play an important role in the regulation of tumor immune escape through the regulation of MDM2 (Fig. 4).

### Immunogenicity of MDM2

The overexpression of proteins in tumors may provide opportunities for specific T-cell responses because a threshold antigen level is required for T cell recognition. If tumor cells present a certain amount of the peptide-HLA complex above the threshold, a specific antitumor T-cell response may occur.^86^ Tumor-associated antigens are autoantigens encoded in the germline genome that are preferentially expressed in tumors and usually weakly immunogenic owing to their central and peripheral tolerance.^87^ As a tumor-associated antigen, MDM2 is overexpressed in most tumors, presents low expression in normal tissues, and is often associated with poor tumor prognosis.^88^^,^^89^ MDM2-overexpressed tumor cells may elicit adaptive immune-mediated T cell killing, whereby the use of tumor antigenic peptides to create tumor vaccines may be efficacious.

Multiple experiments have shown that MDM2 peptides bound to MHC molecules are lifted to T cells by DC antigens, further inducing CD8^+^/CD4^+^ cytotoxic T lymphocyte activation.^90^, ^91^, ^92^ In chronic lymphocytic leukemia, the MDM2-derived peptide MDM2_81-88_ has been shown to elicit an autonomous immune response in CD8^+^ T cells.^90^ However, in patients with invasive squamous cell carcinoma of the head and neck, MDM2-specific expression of CD8^+^ T cells was detected in the blood. This study showed that high MDM2-expressing tumor cells have increased affinity for T cells and directly kill MDM2-overexpressing cancer cells. In addition, the derived peptide MDM2_32-46_ binds to HLA-DR. MDM2 peptides activate CD4^+^ helper T lymphocytes, and activated helper T lymphocytes secrete cytokines such as IFN-γ, activate CTLs, and directly kill tumor cells via granzyme B^92^. These results suggest that MDM2 has the potential to mediate the functional killing of tumor cells by T cells, which could be a valuable research direction in tumor therapy.

Notably, in tumor-bearing mice, two highly conserved MHC I-binding motifs within MDM2, MDM2_441_ and MDM2_100_, elicit anti-tumor responses. MDM2_100_ induces CTL killing in endogenous MDM2-expressing tumors, whereas MDM2_441_ induces less proliferation and differentiation of CTLs. The combination of these two peptides induces a stronger tumor-killing effect, suggesting that a single MDM2 peptide has a poorer effect on tumor suppression than peptides targeting multiple sites.^91^ However, the effect of MDM2_100_ stimulating CTLs disappeared after a certain period of sustained action. This may be the reason for activation-induced cell death in T cells, where activated CTLs exercise effector functions, after the exercise of their effector functions. This also suggests that immunization with MDM2 peptide alone cannot completely kill tumors and that its combination with other therapeutic modalities could be a more attractive treatment approach.^93^, ^94^, ^95^

Tumor vaccines are most commonly administered using MHC I-restricted peptide epitopes that share tumor-associated antigens, which activate specific T cells against self-antigens.^96^ Treatment with ICIs relies on the presence of T cells in the TME, although in many tumor tissues, the number of T cells is low or “depleted”. Treatment with ICIs is clinically ineffective for “cold tumors”. The use of synthetic peptides derived from tumor antigens as cancer vaccines can stimulate the body to produce T cells against tumor antigens. Therefore, the combination of tumor vaccines and ICIs may be one of the directions of tumor therapy in the future.

### Regulation of PD-L1

PD-L1 is an essential protein for the maintenance of immune homeostasis, and the PD-1/PD-L1 pathway physiologically inhibits immune cell overactivation and prevents autoimmune diseases.^97^ However, PD-L1 expression is elevated in many types of malignancies, and the PD-1/PD-L1 axis is hijacked by tumor cells in the TME to evade immune surveillance, thereby facilitating tumor immune escape.^98^ PD-L1 overexpressed in tumor cells binds to PD-1 on tumor-infiltrating lymphocytes, which counteract T-cell receptor signaling by phosphorylating SHP-2, resulting in blocked T cell activation.^99^^,^^100^ Several other cell types in the TME, such as macrophages, DCs, activated T cells, and cancer-associated fibroblasts, also express PD-L1.^101^ These components orchestrate an immunosuppressive microenvironment and support tumor growth. PD-L1 expression is commonly associated with poor prognosis and can be used to predict the efficacy of anti-PD-1/PD-L1 therapy.^102^, ^103^, ^104^ PD-L1 may promote tumor cell proliferation and progression and participate in the regulation of epithelial–mesenchymal transition.^105^^,^^106^ In addition, PD-L1 expression is associated with anti-tumor therapy resistance. All the above factors may contribute to a poor prognosis.^107^

Evidence suggests that PD-L1 expression in cancer cells is mediated by the activation of oncogenic signaling pathways and factors in the TME.^108^ In pancreatic cancer, myeloid cells induce PD-L1 expression in tumor cells through the activation of the EGFR-MAPK pathway.^109^ Similar to the MAPK pathway, the PI3K/A kt pathway may regulate PD-L1 expression in a cell- or tissue-type-specific manner, either through transcriptional or post-transcriptional mechanisms.^110^ Additionally, transcription factors such as HIF-1, STAT3, and NF-κ;B may promote PD-L1 expression. Notably, a growing number of studies have reported a positive correlation between p53 and PD-L1 expression on the surface of tumor cells.^111^, ^112^, ^113^ In one study, the restoration of p53 activity in p53-deficient triple-negative breast cancer cells resulted in the up-regulation of PD-L1 expression on the surface of tumor cells and sensitized them to anti-PD-1 therapy.^114^ These results indicate that p53 plays an important role in the regulation of PD-L1.^115^, ^116^, ^117^

MDM2, as a major p53 regulatory factor, can regulate the expression of PD-L1 in tumor cells, such as melanoma through the p53-dependent MDM2 pathway, and the MDM2 inhibitors Nutlin-3 and APG-115 induce PD-L1 expression on the surface of tumor cells in a p53-dependent manner. Among them, APG-115 regulates PD-L1 expression by up-regulating the STAT3 signaling pathway in mouse hepatocellular carcinoma cells, which further promotes the activity of effector T cells and ultimately enhances the anti-tumor effects of PD-1 inhibitors (Fig. 4).^34^^,^^118^^,^^119^

Existing MDM2 inhibitors promote PD-L1 expression in tumor cells through a p53-dependent pathway, and it is possible to treat tumors in combination with ICIs in terms of molecular mechanisms. Molecules in tumor cells can also regulate the p53-dependent MDM2 pathway. Ubiquitin-specific protease 7 (USP7) regulates the stability of a plethora of intracellular proteins involved in the suppression of anti-tumor immune responses, and its overexpression is associated with poor survival in many cancers. USP7 disrupts the equilibrium of the p53-dependent MDM2 axis, leading to the proteasomal degradation of p53 and ultimately decreasing the expression of PD-L1 on the surface of tumor cells.^120^, ^121^, ^122^ Several USP7 inhibitors are currently in preclinical trials and further studies are required. In addition, high expression of the long non-coding RNA MEG3 can inhibit tumorigenesis and progression through multiple mechanisms.^123^ In esophageal cancer cells, FOXP3 expression is inhibited by the up-regulation of miR-149-3p through p53-dependent MDM2.^73^ FOXP3 can activate PD-L1 transcription, directly inhibit the activity of CD8^+^ T cells in the TME,^124^ and directly activate FOXP3^+^ Tregs in the CCL5-recruited TME.^125^ The levels of CD8^+^ T cells and FOXP3^+^ Tregs in the TME significantly affect the survival of tumor cells, suggesting that the high expression of MEG3 plays an important role in tumor immune escape.

High levels of PD-L1 may suggest a worse prognosis for patients with tumors, which may result from PD-L1-induced inhibition of T-cell activation. However, PD-L1 can also be used as a marker of immunotherapy efficacy, and the use of MDM2 inhibitors to increase PD-L1 expression in tumor cells or the TME in animal experiments with improved immunotherapy efficacy is a seemingly attractive therapy. However, the advantages and disadvantages of high PD-L1 expression still need to be carefully considered to benefit patients with tumors. USP7 and MEG3 have been found to regulate PD-L1 expression through a p53-dependent pathway, but the exact mechanism is not well understood. USP7 inhibitors have been shown in some preclinical experiments to overcome drug resistance in small-cell lung cancer cells^126^ but have not yet reached the clinical study stage. Treatments associated with MEG3 have not been established; therefore, its clinical value is unclear, and further studies are needed.

### Regulation of the MHC

Human MHC is encoded by the HLA gene localized on chromosome 6, and classical MHC-I and MHC-II molecules play key roles in antigen presentation and recognition by T cells.^127^ MHC-I is expressed in almost all nucleated cells, and CD8^+^ T cells recognize tumor cells by recognizing peptide MHC-I complexes generated by the MHC-I antigen presentation pathway and killing these cells via perforin- or FAS-dependent pathways.^128^ Transcription factors, such as NLRC5, IRF1, and IRF2, regulate basal and IFN-induced MHC-I expression. However, MHC-I is not required for cell survival.^129^, ^130^, ^131^, ^132^ Therefore, some tumors can down-regulate or lose MHC-I in response to selective pressure from CD8^+^ T cells and ultimately undergo immune escape, termed immunoediting.^133^^,^^134^ MHC-II is mainly expressed by specialized antigen-presenting cells, such as DCs, B cells, and macrophages, as well as some tumor cells, and primarily presents exogenous peptide antigens to CD4^+^ T cells. Although cytotoxic CD8^+^ T cells are thought to be the main effector cell type in the action of ICIs, CD4^+^ T cells play a key role in supporting CD8^+^ T cell activation and memory T cell production and are now recognized as essential for an effective response to ICIs.^135^, ^136^, ^137^, ^138^, ^139^, ^140^, ^141^, ^142^, ^143^ Owing to the importance of the MHC-mediated immune killing of tumors, the down-regulation of MHC expression by tumors has become an important means of immune escape. Multiple pathways exist in tumor cells that may interact with MHC-II and regulate its expression. Common pathways such as JAK/STAT signaling up-regulate tumor MHC-II expression, whereas, in breast cancer, RAS/MAPK signaling inhibits MHC-II expression.^144^, ^145^, ^146^ MDM2 is overexpressed in a variety of tumors, including melanoma,^147^ and plays an important role in the regulation of MHC expression in tumor cells. MDM2 degrades p53 in melanoma cells to block the expression of CIITA, a key regulator of MHC-II, with a consequent decrease in the levels of MHC-II and IL-15, which leads to decreased CTL and NK cell activation.^49^^,^^92^^,^^148^ Conversely, the inhibition of MDM2 up-regulates the expression of IL-15, MHC-II, and tumor necrosis factor-related apoptosis-inducing ligand receptor-1 and -2 in acute myelogenous leukemia cells and activates CD8^+^ T cells and NK cells, which enhances the anti-tumor effects of T cells within the TME.^50^^,^^149^

Notably, MDM2 is a tumor-specific MHC-II (TsMHC-II), and several studies on various cancer types have found an association between TsMHC-II and a good prognosis.^150^^,^^151^ Further studies found that TsMHC-II was associated with higher numbers of CD4^+^ and CD8^+^ tumor-infiltrating lymphocytes, absence of lymphovascular invasion, formation of tertiary lymphoid structures, up-regulation of genes associated with the activation of the IFN-γ pathway (including CD274, which encodes PD-L1), and higher levels of IFNG, IL-2, and IL-12 mRNA (Th1 cytokines).^152^, ^153^, ^154^ TsMHC-II has been extensively studied in mouse models, and most studies have shown that ectopic MHC–II– or CIITA-transduced tumor cells increase immune-mediated tumor rejection.^155^, ^156^, ^157^, ^158^ Examples of increasing the expression of tsMHC-II in tumors include the use of inhibitors of MDM2, which may allow tumor cells in an immune escape state to be recognized again by T cells and can be combined with ICIs to produce better efficacy.

Intracellular MHC-I synthesis depends on the transporter associated with antigen processing 1 and the downstream endoplasmic reticulum aminopeptidase 1. Both these components are regulated by p53, and the loss of p53 results in impaired MHC-I synthesis (Fig. 4).^159^^,^^160^ In addition, the deletion of MHC-I expression, which is also mediated by the deletion of β2M, has been reported as a mechanism of resistance to treatment with ICIs.^161^^,^^162^ MDM2 inhibitors can potentiate the activity of transporter associated with antigen processing 1 and endoplasmic reticulum aminopeptidase 1 through a p53-dependent pathway, which ultimately leads to an increase in MHC-I expression in tumor cells,^163^ triggering a subsequent T-cell anti-tumor response.

Down-regulation of MHC is an important means for tumors to achieve immune escape and is one of the causes of immunotherapy resistance. MDM2 inhibition can also modulate the expression of MHC-I and MHC-II in tumor cells in different ways, which seems to suggest that MDM2 inhibition is an excellent way to avoid resistance to ICIs.

## MDM2 and hyperprogressive disease during immunotherapy

Immunotherapy has significantly enhanced the treatment of patients with metastatic tumors.^164^ However, in a 2016 phase I clinical study of PD-1/PD-L1 inhibitors in tumors, Champia first reported the existence of a new highly progressive and aggressive pattern under immunotherapy and showed that tumors that grow drastically after PD-1/PD-L1 inhibitor treatment can increase in volume by up to 20–30-fold and have a poor prognosis, which is termed hyperprogressive disease (HPD). Champia first reported an HPD incidence of 9% (12/131), which was independent of baseline tumor load or tumor type, and an even higher incidence was observed in patients aged more than 65 years.^165^ Several subsequent studies have reported the use of ICIs alone for malignant solid tumors, with a response rate of approximately 20%–40%.^166^, ^167^, ^168^ Kato et al defined HPD as follows: rate of tumor progression (tumor growth kinetics ratio) > two-fold, percentage of RECIST (increase in tumor load during ICIs) > 50%, and time to treatment failure <2 months.^169^

Furthermore, studies on HPD have analyzed somatic genetic alterations in four patients with HPD using next-generation sequencing and found that MDM2 gene amplification was detected in 50% of the patients with HPD.^170^ Another study found that although no statistically significant association was observed between MDM2 amplification and the efficacy of ICIs, it was still observed that patients with MDM2 amplification were more likely to develop HPD and had lower disease control rates.^171^ Further, some investigators have found that MDM2 overexpression is frequently detected in B-cell lymphomas, while mutations in P53 occur infrequently. In the proliferation of B cells in germinal centers, IFN regulatory factor 8 binds to the MDM2 promoter P2, resulting in increased transcription of MDM2, which promotes B-cell expansion by degrading p53 and may mediate lymphoma generation.^172^ MDM2 overexpression in Burkitt's lymphoma is mainly associated with post-transcriptional modifications and is not caused by MDM2 gene.^173^ MDM2 overexpression increases B cell proliferation and decreases apoptosis through increased genomic instability and via p53, leading to the development of B cell lymphomas.^174^ MDM2 binding protein binds to the finger ring structure of MDM2 and increases the ubiquitination of MDM2 to degrade p53.^175^ In other studies, its up-regulation was observed in MYC-induced lymphomas; however, it was not implicated in the promotion of lymphoma.^176^ Rather, MYC inhibits apoptosis and promotes lymphoma development by inactivating the AFR-MDM2-p53 axis.^177^ Therefore, a further understanding of the molecular mechanisms through which MDM2 promotes HPD has become an urgent issue for improving immunotherapy and preventing HPD.

ICIs mainly bind to CTLA-4 and PD-1 receptors on the surface of T cells and to PD-L1 receptors on the surface of tumors to relieve the immunosuppression of T cells, thus killing tumor cells.^178^ Wang et al compared the tumor tissues of patients with MDM2 mutations with those of patients without the mutation using next-generation sequencing and found that CD8^+^ T cells, CD68^+^ HLA-DR-M1 macrophages, CD68^+^ HLA-DR-M2 macrophages, and CD56^bright^ and CD56^dim^ NK cells were reduced to different degrees, with CD56^dim^ NK cells showing the greatest difference.^179^ Wang et al showed that MDM2 could significantly affect the infiltration level of immune cells in tumor tissues and cytokine secretion, thereby affecting the therapeutic effects of ICIs; however, the specific mechanism was not elucidated. Furthermore, in several recent studies, overexpression of MDM2 may regulate downstream endogenous retrovirus and miR-149-3p levels through a p53-dependent pathway, and ubiquitination modification regulates FOXP3 expression, which further increases CD25 and CTLA-4 expression on the surface of Tregs and decreases their secretion of IL-2, IL-4, IL-10, and IFN-γ.^32^^,^^72^^,^^73^ Overexpression of MDM2 can also reduce T cell activation by degrading the transcription factor NFATc2 in the nucleus of naïve CD4^+^ T cells via a p53-independent pathway.^35^^,^^180^

In addition, the TME is usually hypoxic, and it has been shown that hypoxia leads to immune evasion and therapeutic resistance of tumors to ICIs.^181^ Whereas under stress conditions such as hypoxia, MDM2 translocates from the nucleus to the cytoplasm of neuroblastoma cells via p53-independent MDM2 signaling pathway and binds to AU-rich elements within the VEGF mRNA 3′ UTR. This binding to the VEGF 3′ UTR increases the stability of VEGF mRNA and increases VEGF translation, thereby significantly enhancing its production, ultimately leading to tumor growth and angiogenesis.^182^ Moreover, JAK-STAT signaling activated by ICIs can increase the IFN regulatory factor IRF-8, which binds to the MDM2 promoter and induces the expression of MDM2.^183^ The overexpression of MDM2, in turn, can diminish the efficacy of immunotherapy and promote tumor growth in the ways mentioned above, which may ultimately lead to hyperprogression of immunotherapy through multiple pathways.

In conclusion, MDM2 overexpression is an essential factor in the induction of HPD, which not only regulates the activation of T cells and cytokine secretion in the tumor immune microenvironment to reduce the efficacy of ICIs but also promotes tumor growth. Therefore, targeting MDM2 may be an important strategy for the prevention of HPD.

## MDM2 inhibitors modulate the TME

In the previous section, we discussed the regulatory mechanisms of MDM2 in various types of immune cells in the immune microenvironment. Correspondingly, MDM2 inhibitors also regulate the tumor immune microenvironment, mainly by affecting the p53-dependent MDM2 pathway. However, because of the different drug structures of different MDM2 inhibitors, their modes of action and regulation in the immune microenvironment may vary. The mechanisms by which various MDM2 inhibitors modulate the tumor immune microenvironment are summarized below, and the functions of the MDM2 inhibitors mentioned in this article are summarized in Table 1.

**Table 1:** Biological properties of the selected MDM2 inhibitors with demonstrated effect on anti-tumor immune response.

Molecule name	Chemical structure	Key features	Cancer type in vitro and/or in vivo studies	Immune function
Navtemadlin (KRT-232, AMG-232)		Piperidinone	NSCLC, Merkel Cell Carcinoma， Ovarian Clear Cell Carcinoma	T cell activation ↑
Siremadlin (HDM-201)		Pyrrolidonoimidazole scaffold	Colon Cancer, Melanoma	DC proliferation ↑ T cell activation ↑ MHC expression ↑ NK cell apoptosis↓
Alrizomadlin (APG-115)		Spirooxindole based	Melanoma, MPNST, NSCLC, Solid Tumors, Well Differentiated/Dedifferentiated Liposarcoma	M1-Macrophage polarization↑ PD-L1 expression↑
Nutlin3		Cisimidazoline analogues	Lymphoma, Melanoma	DC and macrophage proliferation↑ T cell activation ↑ PD-L1 expression↑
RG7112		Cisimidazoline derivative	Melanoma	T cell activation↑ MHC expression↑

Nutlin-3 activates antigen-presenting cells (dendritic cells, macrophages, *etc*.) through the p53-dependent MDM2 pathway to promote the proliferation of lymphocytes, including CD4^+^ and CD8^+^ T cells. It also increases the levels of cytokines, such as IL-12 and p40, to regulate immune responses and further activate tumor immunogenic cell death in the TME, leading to tumor regression.^64^^,^^184^^,^^185^ However, other studies have found that Nutlin-3 induces PD-L1 and CD276(B7–H3) expression on the surface of melanoma cells in a p53-dependent MDM2 manner.^119^ PD-L1 mediates CD4^+^ T cell suppression, whereas CD276, a recently discovered immune checkpoint, has dual effects. On the one hand, it inhibits T cell activity and reduces TME T cell infiltration by down-regulating NF-κB, NFAT, and AP-1 signaling pathways, thereby leading to an immunosuppressive TME.^186^ On the contrary, it promotes tumor anti-apoptosis and growth through the JAK/STAT and PI3K/Akt/mTOR pathways.^187^^,^^188^

Similarly, HDM201 mediates an increase in the percentage of mouse DCs (including the CD103^+^ antigen cross-presenting sub population), as well as an increase in the percentage of T bet^+^ Eomes^+^ CD8^+^ T cells and the CD8^+^/Treg ratio within tumors through the p53-dependent MDM2 pathway. Enhanced CD80 expression was observed in tumor cells, which further stimulated the value of T cells, especially CD8^+^ T cells, in the CD45^+^ population and elevated PD-L1 expression.^33^ In addition, a study found that MDM2 inhibitors mediate an increase in the expression of IL-15 and MHC-II in mouse melanoma cells via the p53-dependent MDM2 pathway.^50^ MDM2 inhibitors not only promote IL-15 production by cancer-associated fibroblasts, macrophages, and B cells but also increase IL-15 production by melanoma cells, with high IL-15 expression positively correlated with mouse viability. Melanoma cells expressing MHC-II are susceptible to elimination by tumor-specific cytotoxic CD4^+^ T cells, leading to tumor regression in an adoptive T cell transfer model.^143^^,^^189^ Additionally, MHC-II expression in human melanoma cells has been associated with response to anti-PD-1 therapy.^151^^,^^190^ However, studies have also reported an immunosuppressive effect of MHC-II expression on melanoma cells.^191^

*In vitro* treatment of bone marrow-derived macrophages with APG-115 resulted in the activation of p53 and p21 and a reduction in the immunosuppressive M2 macrophage population through the down-regulation of c-Myc and c-Maf. Increased polarization of proinflammatory M1 macrophages was observed in the spleens of mice treated with APG-115. In addition, APG-115 exhibits co-stimulatory activity in T cells and increases PD-L1 expression in tumor cells.^34^

Preclinical experiments with MDM2 inhibitors have shown paradoxical results because they can enhance anti-tumor effects by promoting T lymphocyte proliferation and M1 macrophage polarization, which are inhibited by promoting immune checkpoint expression. Current experimental evidence suggests that most of these functions are mediated by p53 activation. Moreover, p53 may modulate PD-L1 levels through miR-34.^117^ Although most preclinical experiments with MDM2 inhibition have demonstrated tumor suppression, the impact of PD-L1 up-regulation should be determined in further clinical trials. Additionally, PD-L1 levels are predictive of immunotherapeutic efficacy, and combining MDM2 inhibitors with ICIs seems feasible.

## MDM2 inhibitors combined with ICIs

Since the first-generation MDM2 inhibitor Nutlin was developed in 2004, several dozen MDM2 inhibitors have been developed, nine of which have entered clinical trials.^192^ Despite demonstrating potent anti-tumor cell proliferation effects in preclinical experiments, the clinical efficacy of MDM2 inhibitors has been somewhat disappointing. For instance, in clinical trials on leukemia treatment, compounds such as RG7112 and RG7388 (idasanutlin) failed to induce significant tumor responses in most patients.^193^^,^^194^ However, tumor resistance poses a significant limitation to the efficacy of MDM2 inhibitors. When excess Nutlin is introduced into wtp53 lymphoma cells, the tumor cells quickly develop resistance to Nutlin. Furthermore, they exhibit cross-resistance to drugs such as bortezomib, doxorubicin, cisplatin, and methotrexate. However, subsequent gene sequencing revealed that MDM2 did not undergo mutations, whereas mutations were identified in the p53 DNA-binding region and the dimerization structural domain.^195^ One theory is that p53 mutations are inherently present in these tumors and only detected because of the growth advantage of mutant tumor cells owing to drug selection pressure. However, further studies have found that MDM2 inhibitors are more likely to cause mutations in intracellular p53 by inducing and/or promoting DNA damage.^196^ To overcome tumor resistance, researchers have begun clinical attempts to combine MDM2 inhibitors with chemotherapy. Most of these studies are still in the experimental phase. However, some studies have been unsuccessful, as shown by the results of an investigation of idasanutlin in combination with cytarabine for the treatment of relapsed or refractory acute myelogenous leukemia, in which the combination did not improve overall survival or complete response rates, compared with cytarabine plus placebo (median survival 8.3 *vs*. 9.1 months; hazard ratio = 1.08; 95% confidential interval = 081–1.45; *p* = 0.58).^197^ Notably, mutant P53 can increase the tumor mutational load by blocking DNA damage repair and inactivating cell cycle checkpoints. It can serve as a predictor of ICI therapy effectiveness; moreover, p53 mutation-associated genome instability plays a positive role in ICI therapy.^198^, ^199^, ^200^ ICIs represent a popular oncological treatment modality in recent years and have shown improved efficacy in melanoma and other cancers, although the effective rate for all tumors is still insufficient.^201^ A xenograft melanoma model study showed that the local activation of wtp53 with Nutlin-3 overcame immunosuppression and enhanced anti-tumor immunity by inducing immunogenic cell death.^202^ These findings indicate that combination therapy with MDM2 inhibitors and ICIs may have complementary effects, thereby enhancing therapeutic efficacy.

Therapeutic combinations with ICIs have already been demonstrated in several animal studies. In one study, the addition of pembrolizumab in combination with AMG-232 to a T cell and tumor cell co-culture system resulted in enhanced T cell-mediated killing of tumor cells and was expected to be translationally useful in the clinic.^28^ In another study, MDM2 inhibitors combined with anti-PD-1 antibodies in melanoma-bearing mice resulted in improved survival and fewer lung metastases compared with the treatment of MDM2 inhibitors alone. Further analysis revealed that MDM2 inhibitors promoted the frequency of CD8^+^ CD73^+^ TCF-1^+^ IFN-γ^+^ TNF^+^ T cells and T-cell receptor activation and that isolation of these T cells from mice and transfer to PD-1-treated mice increased survival and activated immune memory against melanoma cells.^50^ While these animal studies undoubtedly provide a theoretical basis for formal clinical trials, the results of a recent phase Ib/II clinical study on MDM2 inhibitors in combination with ICIs are equally encouraging. Regarding the small molecule inhibitor of MDM2 alrizomadlin (APG-115) in combination with pembrolizumab for the treatment of unresectable/metastatic solid tumors that have progressed on immunotherapy in adults or children (NCT03611868), preliminary and interim findings from 6 cohorts encompassing 130 patients reveal the following outcomes: a 13% overall response rate in melanoma, with 2 complete responses and 3 partial responses among 38 patients with assessable efficacy. Within the cutaneous and uveal melanoma subgroups, overall response rates of 24% (2 complete responses + 2 partial responses/17 assessable efficacy patients) and 9% (1 partial response/11 assessable efficacy patients) were observed, respectively. Malignant peripheral nerve sheath tumors exhibited a clinical benefit rate of 40% (4 stable diseases/10 assessable efficacy patients, defined as overall response rate + standard deviation > 4 cycles). Additionally, one case of partial response was documented for each patient with non-small cell lung cancer, urothelial cancer, and liposarcoma. The results demonstrated that the combination therapy was well tolerated, showed preliminary anti-tumor activity in a wide range of tumor types, and restored anti-tumor effects in cancer patients who were resistant or intolerant to immunotherapy.^203^ In addition, several other clinical studies (Table 2; NCT03787602, NCT05705466, NCT03964233, NCT03940352, NCT03555149, NCT03566485, NCT04785196, NCT03611868, and NCT05447663) have been initiated and are currently recruiting participants. We look forward to the results of these clinical studies to further advance the clinical applications of MDM2 combination immunotherapy.

**Table 2:** Clinical trials of MDM2 inhibitors combined with immunotherapy.

NCT Number	Diseases	Phase	Intervention 1	Intervention 2	Population	Status
NCT03787602	Merkel Cell Carcinoma	1,2	Navtemadlin (KRT-232)	Navtemadlin + Avelumab(anti PD-L1)	Enrollment:115 Age:18 Years and older Sex:All	Recruiting
NCT05705466	Non Small Cell Lung Cancer	1,2	Navtemadlin + Pembrolizumab(anti PD-1)	Navtemadlin placebo + Pembrolizumab	Enrollment:92 Age:18 Years and older Sex:All	Not yet recruiting
NCT03964233	Solid Tumors	1,2	Dose Escalation/Expansion-BI 907828 +Ezabenlimab(anti PD-1)	Dose Escalation-BI 907828 + Ezabenlimab + BI 754111(anti LAG-3)	Enrollment:140 Age:18 Years and older Sex:All	Recruiting
NCT03940352	Acute Myeloid Leukemia, High-risk Myelodysplastic Syndrome	1	Siremadlin(HDM201)+ MBG453(anti TIM-3)	Siremadlin + Venetoclax	Enrollment:52 Age:18 Years and older Sex:All	Active, not recruiting
NCT03555149	Colorectal Cancer	1,2	Idasanutlin(RG7338)+ Atezolizumab(anti PD-L1)	Atezolizumab + other treatment (Imprime PGG + Bevacizumab, Isatuximab, Selicrelumab + Bevacizumab, Regorafenib, Regorafenib + AB928, LOAd703)	Enrollment:96 Age:18 Years and older Sex:All	Terminated
NCT03566485	Stage III and IV (ER+) Breast Cancer	1,2	Idasanutlin(RG7338) + Atezolizumab	–	Enrollment:12 Age:18 Years and older Sex:Female	Terminated
NCT04785196	Liposarcoma, Advanced Solid Tumor	1,2	Alrizomadlin(APG-115)+ Toripalimab(anti PD-1)	–	Enrollment:95 Age:18 Years and older Sex:All	Recruiting
NCT03611868	Unresectable or Metastatic Melanoma, Advanced Solid Tumors	1,2	Alrizomadlin(APG-115)+ Pembrolizumab	–	Enrollment:140 Age:12 Years and older Sex:All	Recruiting
NCT05447663	Acute Myeloid Leukemia	1,2	Siremadlin(HDM201) + Donor Lymphocyte Infusion	–	Enrollment:38 Age:18 Years and older Sex:All	Recruiting

Notably, the toxicity of MDM2 inhibitors has raised concerns in clinical studies. Evidence from both animal and cellular experiments indicates the toxic effects of MDM2 inhibition on normal development and cellular homeostasis. For instance, MDM2 deficiency leads to early embryonic lethality in mice, and MDM2-deficient cells cannot survive in culture.^204^^,^^205^ Prominent myelosuppressive toxicity has been demonstrated in further clinical studies. The first phase I single-agent study of milademetan in patients with advanced liposarcoma, solid tumors, or lymphoma (NCT01877382) administered milademetan orally once daily for 28 days in both extended (days 1–21) and continuous (days 1–28) dosage schedules, with a starting dose of 15 mg. The results showed an incidence of 69.6% and 36.2% (*n* = 569) for all grades and grade 3/4 drug-associated thrombocytopenia in the extended/continuous dosing regimen, respectively, with 16 (23.2%) and 24 (34.8%) patients requiring dose reduction or discontinuation of the drug owing to thrombocytopenia and 8 (11.6%) patients experiencing serious adverse events.^206^ Similarly, in the context of combination immunotherapy, a study involving alrizomadlin in combination with pembrolizumab for the treatment of adults or children with immune therapy-refractory/unresectable metastatic solid tumors (NCT03611868) revealed 3/4-grade drug-related adverse events (≥5%, *n* = 130) that included 30 cases of thrombocytopenia (22.9%), 13 cases of neutropenia (10%), and 9 cases of anemia (6.9%). Six patients (4.6%) discontinued treatment owing to treatment-related adverse events, including 3 cases of grade 4 thrombocytopenia. Additionally, 10 patients (7.7%) experienced treatment-related serious adverse events.^202^ Myelosuppression by MDM2 inhibitors may be attributed to the fact that p53 is a component of the self-regulatory loop in hematopoietic stem cells and can promote the apoptosis of megakaryocyte progenitor cells. Once activated by MDM2 inhibition, it hinders platelet production.^207^ Some investigators have mitigated the myelosuppressive toxicity of MDM2 inhibitors using intermittent dosing; however, their overall adverse effects remain prominent. Interestingly, a recent USP2 inhibitor was found to activate p53 function and maintain the integrity of the p53-dependent MDM2 axis, which inhibits tumor growth in mice without exhibiting significant toxicity, similar to MDM2 inhibitors.^208^ This seems to point to a new direction for targeting p53 for tumor treatment in the future.

In conclusion, MDM2 inhibitors in combination with ICI therapy can improve anti-tumor effects and tumor-overcoming resistance when MDM2 monotherapy is not ideal, and combination therapy does not appear to increase MDM2 inhibitor toxicity; however, further clinical trials are needed to validate these findings.

## Conclusion and perspective

MDM2 is a widely recognized suppressor of p53. However, recent studies have revealed a connection between MDM2 dysregulation, tumorigenesis, and immune evasion. This comprehensive review delineates the role of MDM2 in the regulation of innate and adaptive immunity within the TME. Notably, MDM2 governs the dysfunction or depletion of CD8^+^ T cells through both p53-dependent MDM2 and p53-independent MDM2 signaling pathways. Moreover, it augmented the activities of both CD8^+^ and CD4^+^ T cells. This dual functionality underscores the intricate regulatory mechanisms and reveals that further investigation of the underlying processes is required. MDM2 inhibits the anti-tumor activity of NK cells and DCs. It facilitates the differentiation and proliferation of Tregs while promoting the polarization of TAMs, primarily shifting them toward an M2-like macrophage phenotype. Collectively, these actions contribute to the immunosuppressive milieu in the TME. Immunosuppression orchestrated by MDM2 plays a crucial role in tumor metastasis, drug resistance, and immune evasion. Furthermore, MDM2 induces the expression of immunosuppressive molecules or their corresponding receptors, such as PD-L1/PD-1 and MHC, on the cell membranes of both tumor and immune cells to mediate tumor immune evasion. Although ICIs have led to significant advances in anti-tumor therapy, a subset of patients develop HPD after ICI treatment. This result may be closely related to MDM2 amplification, which regulates the level of immune cell infiltration and cytokine secretion in tumor tissues and thus can promote tumor growth and angiogenesis.

Most human tumors retain the wtp53.^209^ However, functional inactivation of p53 occurs, rendering it an ineffective tumor suppressor. Therefore, determining whether MDM2 inhibitors can revive the latent tumor-suppressive capabilities of p53 represents a novel avenue for anti-tumor therapy. Although preclinical studies have yielded promising results for MDM2 inhibitors, the efficacy of these inhibitors in clinical trials has been limited and is accompanied by significant drug-related adverse effects. Combining MDM2 inhibitors with immunotherapy to overcome resistance to ICIs is a good choice. A preliminary clinical study of ICIs combined with MDM2 inhibitors showed that combination therapy can overcome tumor resistance and immune escape, and the safety and tolerability of the combination therapy.^202^ However, further clinical studies are required to confirm these findings.

In conclusion, MDM2 inhibitors combined with immunotherapy have therapeutic potential in anti-tumor therapy, but results from more clinical trials are needed to confirm this conclusion.

## Author contributions

QRZ, SCZ, and XFD: writing-original draft preparation, corrections of the revised version, review, editing, and preparation of figures and tables. CYH, RWR, and YD: writing-review, editing, and figure preparation. JD, XMT, and JPX: conceptualization, supervision, writing-review and editing, and figure preparation. JD and JPX: funding acquisition.

## Conflict of interests

The authors declared no competing financial interests.

## Funding

Open Access funding was enabled and organized by Project DEAL. This work was supported by the 10.13039/501100001809National Natural Science Foundation of China (No. 82202869, 82260491, 82160459), Jiangxi Province “Double Thousand Program” (China) (No. jxsq 2023201108), 10.13039/501100002858China Postdoctoral Science Foundation (No. 338937), and the Jiangxi Provincial Natural Science Foundation (China) (No. 20232ACB206044).
