# Supplementary material for: MDM2 inhibitors in cancer immunotherapy: Current status and perspective
Source: Genes Dis. 2024 Mar 28;11(6):101279. doi: 10.1016/j.gendis.2024.101279 (PMC11388719; doi:10.1016/j.gendis.2024.101279)
Supplement: Multimedia component 2 [file mmc2.pdf]

After discussion among all authors of the original manuscript, we agreed to add Xiaofeng Dai as co-first author of "MDM2 inhibitors in cancer immunotherapy: current status and perspective".

All authors signed below:

Qinru Zeng, Shaocheng Zeng, Xiaofeng Dai, Yun Ding, Chunye Huang, Ruiwen Ruan,

Qinru Zeng Shaocheng Zeng . Xiaofeng Dai Yun Ding , Chunye Hung Ruiwen Ruam .

Jianping Xiong, Xiaomei Tang, Jun Deng

Jianping Xiong Xiaomei Tang Jun Deng
